# High dose and compartmental target volume may improve patient outcome after radiotherapy for pelvic bone metastases from hepatocellular carcinoma

**DOI:** 10.18632/oncotarget.9767

**Published:** 2016-06-01

**Authors:** Taehyung Kim, Hye Jung Cha, Jun Won Kim, Jinsil Seong, Ik Jae Lee

**Affiliations:** ^1^ Department of Radiation Oncology, Yonsei University College of Medicine, Yonsei University Health System, Seoul, Korea; ^2^ Department of Radiation Oncology, Gangnam Severance Hospital, Yonsei University College of Medicine, Seoul, Korea

**Keywords:** hepatocellular carcinoma, pelvic bone metastasis, radiotherapy, compartmental target volume, local control

## Abstract

**Purpose:**

Pelvic bone metastases are difficult to treat because of complex pelvic bone anatomy and the proximity of normal organs. The adequacy of radiation dose and field coverage was evaluated.

**Patients and methods:**

We analyzed 146 cases of pelvic bone metastases from HCC treated with radiotherapy (RT). Bone metastases were confirmed using CT/MRI. Subjective pain response was assessed using the visual analogue scale, and treatment-related toxicity with the Common Terminology Criteria for Adverse Events v3.0. Local failure free survival (LFFS) and overall survival were estimated using the Kaplan-Meier method.

**Results:**

The local control rate was 80.1% and the pain control rate was 68.5%. Compartmental target volume (CTV), encompassing the whole compartment of the involved bone, was found to be a significant factor (1-year LFFS, 78% vs. 50%; *p*=0.001). Sites of metastasis were categorized as either upper or lower pelvic bone; both categories showed improved local control with CTV. Metastatic lesions that received more than 50 Gy of EQD2 showed more partial response in pain after RT (58% vs. 79%; *p*=0.007). No patient showed toxicity higher than Grade IV.

**Conclusion:**

Compartmental RT targeted to the involved bone was associated with improved local control and LFFS. High-dose radiation was associated with an improved treatment response.

## INTRODUCTION

The bone is a major site of extrahepatic metastasis from hepatocellular carcinoma (HCC). Patients with terminal Barcelona-Clinic Liver Cancer stage D HCC require full symptomatic palliation for local disease or distant metastasis.[1] Palliative radiotherapy (RT) effectively manages symptoms in HCC patients with bone metastases, with pain response rates of approximately 60-80%.[2–4] Pain, bone destruction causing mechanical instability, and pathological fractures are the most common manifestations of bone metastases. To date, several studies described clinical features and radiotherapeutic strategies in HCC patients with bone metastasis, and many studies have evaluated stereotactic body RT for spine metastasis.[5–8] Unlike vertebral metastases, few studies have evaluated HCC patients with pelvic bone metastasis. The pelvic skeleton supports the balance of the trunk and has a complex bone anatomy. Many organs such as the intestines, urinary bladder, and internal sex organs are located within or near the pelvis. With these considerations, RT planning for pelvic bone metastases is frequently challenging.

We analyzed the clinical outcomes including pain palliation and local control according to clinical features and radiotherapeutic parameters in HCC patients who received RT for pelvic bone metastases.

## RESULTS

### Patients

A total of 146 bone metastases from 89 patients were analyzed. Median patient age was 56 years (range, 36-79 years), and 70 patients (79%) were male. The most common viral background was hepatitis B virus (n=77; 86%), and 71 patients (80%) were classified as Child-Pugh class A. Sixty-five patients (72%) had a controlled primary tumor, while 24 (28%) had an uncontrolled primary tumor. An uncontrolled primary tumor was defined as the presence of viable tumors in the liver or HCC progression (Supplementary Table 1). Bone metastasis (n=146) characteristics are summarized in Table 1. Thirty-two patients had multiple pelvic bone metastases. We categorized the sacrum and ilium as upper pelvic bone, and the acetabulum, pubic bone, and ischium as lower pelvic bone. Of 110 cases with other bone metastasis, the most common site was the T-spine (n=83), followed by the L-spine (n=75), scapulae (n=8), C-spine (n=7), ribs (n=6), sternum (n=5), and skull (n=4). There were 59 out of 146 cases (40%) receiving sorafenib, and there were 24 out of 59 cases (41%) were treated RT and sorafenib simultaneously.

**Table 1:** Bone metastasis characteristics

Variable	Group	n	%
Site	Sacrum	44	30%
	Ilium	43	29%
	Acetabulum	26	18%
	Pubic bone	17	12%
	Ischium	16	11%
Site categorization	Upper pelvic bone	87	67%
	Lower pelvic bone	43	33%
Primary controlled	Controlled	36	25%
	Uncontrolled	110	75%
Number of pelvic bone metastasis	Single	59	40%
	Multiple	87	60%
Other bone involvement	Present	110	75%
	Absent	36	25%
Characteristics	Mixed	136	93%
	Pure osteolytic	10	7%
Soft tissue extension	Present	41	28%
	Absent	105	72%

### Local control, pain control and survival analysis

The median follow-up time was 9.7 months (range, 2.1-42.3 months). The local control rate (LCR) was 80.1%, the pain control rate was 68.5%, and the 1-year pain free interval (PFI) was 70%. The LCR and pain control rate according to bone metastasis characteristics are summarized in Table 2. There were more local failures and recurrences of pain in cases of pure osteolytic lesions than mixed metastasis (*p*=0.004 and 0.002, respectively). Cases of bone metastasis with soft tissue extension had more local failures (*p*= 0.025).

**Table 2:** Local control and pain control

Variable	Group	Local control (%)	p value	Pain control (%)	*p value*
Site	Sacrum (44)	89%		64%	
	Ilium (43)	70%		72%	
	Acetabulum (26)	88%		79%	
	Pubic bone (17)	77%		77%	
	Ischium (16)	82%		68%	
Site categorization	Upper pelvic bone (87)	75%		68%	
	Lower pelvic bone (43)	83%		70%	
Primary controlled	Controlled (36)	75%	0.254	58%	0.097
	Uncontrolled (110)	82%		72%	
No. of pelvic bone metastasis	Single (59)	78%	0.588	71%	0.564
	Multiple (87)	82%		67%	
Other bone involvement	Present (110)	81%	0.683	67%	0.579
	Absent (36)	78%		72%	
Characteristics	Mixed (136)	83%	0.004	72%	0.002
	Pure osteolytic (10)	40%		20%	
Soft tissue extension	Present (41)	68%	0.025	76%	0.247
	Absent (105)	85%		66%	

**Figure 1:**
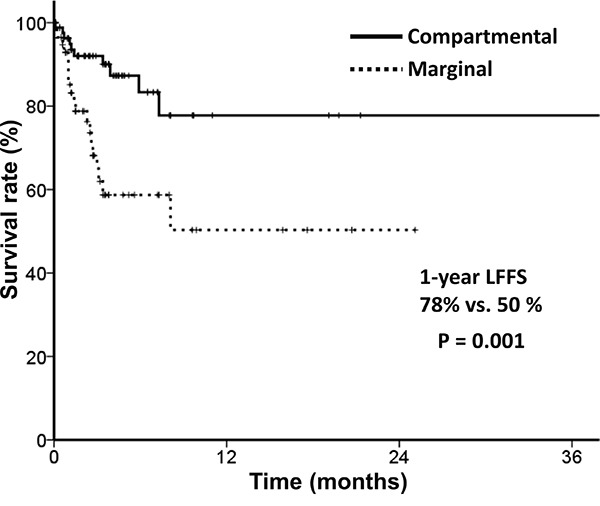
Local failure free survival according to target volume

Local failure free survival (LFFS) regarding clinical target volume (CTV) is depicted in Figure 1. Compartmental target volume was a significant factor for LFFS (1-year LFFS, 78% vs. 50%; *p*=0.001). Bone metastasis characteristics according to CTV are summarized in Table 3. Lesions treated with compartmental target volume were less often classified as Child-Pugh class C (*p*=0.04) and were less osteolytic (not statistically significant). We performed subgroup analysis of bone metastasis characteristics. Sites were categorized into either upper or lower pelvic bone, and both categories showed improved local control with compartmental target volume treatment. Treatment with compartmental target volume showed improved local control over marginal target volume in cases without other bone involvement, soft tissue extension, and with mixed type bone metastasis (Table 4). A trend toward better local control was observed in cases of bone metastasis with primary controlled and single pelvic bone metastasis. Regarding pain control, however, no difference was detected between compartmental and marginal target volumes (Supplementary Table 2).

**Table 3:** Bone metastasis characteristics according to target volume

Variable		Compartmental (n=85) n (%)	Marginal (n=61) n (%)	*p* value
Child-Pugh class	A	64 (75%)	46 (75%)	0.04
	B	17 (20%)	6 (10%)	
	C	4 (5%)	9 (15%)	
Primary controlled		23 (27%)	13 (21%)	0.276
Single pelvic bone metastasis		36 (42%)	23 (38%)	0.572
Other bone involvement		66 (78%)	44 (72%)	0.446
Soft tissue extension		25 (29%)	16 (26%)	0.673
Metastasis characteristic	Osteolytic	3 (4%)	7 (12%)	0.06

**Table 4:** Local control according to target volume

Variable	Group	Local control rate (%)		*p* value
		Compartmental	Marginal	
Pelvic subsite	Sacrum (44)	63%	19%	0.38
	Ilium (43)	30%	30%	0.31
	Acetabulum (26)	95%	75%	0.125
	Pubic bone (17)	86%	57%	0.16
	Ischium (16)	89%	50%	0.135
Site categorization	Upper pelvic bone (87)	86%	68%	0.044
	Lower pelvic bone (43)	59%	13%	0.001
Primary controlled	Controlled (36)	61%	39%	0.051
	Uncontrolled (110)	59%	41%	0.371
Number of pelvic bone metastasis	Single (59)	62%	38%	0.06
	Multiple (87)	58%	42%	0.245
Other bone involvement	Present (110)	53%	47%	0.234
	Absent (36)	75%	25%	0.023
Soft tissue extension	Present (41)	60%	40%	0.428
	Absent (105)	61%	39%	0.022
Metastasis characteristic	Mixed (136)	89%	74%	0.023
	Pure osteolytic (10)	67%	29%	0.5

According to pelvic metastasis subsites, treatment with compartmental target volume showed better local control with the exception of iliac bone metastasis. Local control was very poor with both compartmental and marginal target volume in cases of iliac bone metastasis, and no difference in local control was detected between target volumes. Therefore, in cases of iliac bone metastasis, higher dose RT should be considered. For patients with other bone involvement, no difference was detected according to treatment volume. Therefore, treatment volume according to bone marrow volume should be considered.

Because different fractional doses were used, we calculated RT dose as equivalent 2 Gy fractions (EQD2) assuming an α/β ratio of 10 Gy. There were 14 out of 146 cases (10%) received more than 50 Gy of EQD2; sacrum (n=4), ilium (n=6), and lower pelvic bone (n=4). Metastatic lesions that received more than 50 Gy of EQD2 showed more partial response in pain after RT (58% vs. 79%; *p*=0.006).

Effects of targeted agent (sorafenib) were analyzed. Adding systemic treatment had no effect on local control, pain control and survival.

**Figure 2:**
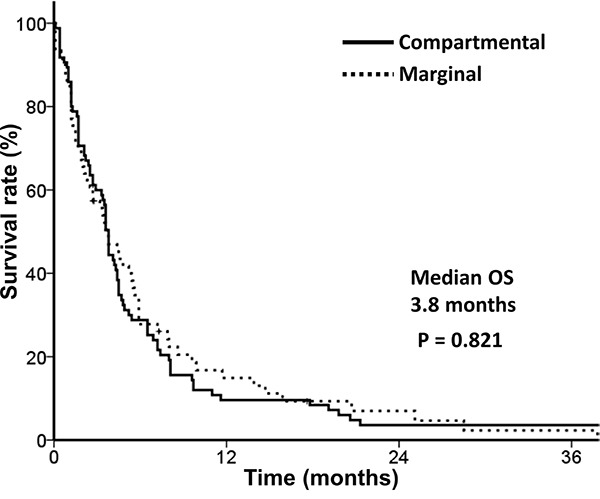
Overall survival according to target volume

Median OS was 3.8 months, and no difference in OS was detected between different CTVs (Figure 2).

### Toxicity

Acute RT-related toxicities are summarized in Table 5. Grade IV neutropenia and thrombocytopenia (n=4 and n=3, respectively) were observed with compartmental target volume treatment. No grade IV toxicities were observed with marginal target volume treatment. However, these were not statistically significant (*p*=0.087 for neutropenia and *p*=0.162 for thrombocytopenia, respectively).

**Table 5:** Toxicity by target volume

Toxicity (Grade)	Compartmental (n=78)				Marginal (n=68)			
	I	II	III	IV	I	II	III	IV
Anemia	9	15	1	0	8	10	4	0
Neutropenia	5	17	7	4	7	12	10	0
Thrombocytopenia	9	10	7	3	7	11	8	0

## DISCUSSION

### The incidence and distribution of extrahepatic bone metastasis

Extrahepatic metastases from primary HCC confer poor prognoses. Frequent sites of HCC metastasis include the lungs (55%), lymph nodes (53%), and bone (28%).[9] The bone is the third most common site of extrahepatic metastasis, and Xiang *et al.* reported that the frequency of bone metastases in 350 HCC patients who had undergone curative resection was 11.7%.[10] Fukutomi *et al.* reported that the incidence of bone metastases from HCC is increasing. Bone metastasis was found in 12 of 269 patients with HCC (4.5%) between 1978 and 1987, and 52 of 404 patients with HCC (12.9%) between 1988 and 1997. More recently, the incidence of bone metastasis has significantly increased (*p*<0.0004). [11] The vertebrae are the most common sites of HCC bone metastasis (68.8%). Thoracic vertebrae are most frequently involved, followed by lumbar and cervical vertebrae. In a study of 203 patients with HCC, He *et al.* reported that the axial skeleton was the most common site of bone metastasis, with metastases occurring most frequently in the spine (46.0%), pelvis (18.5%), and ribs (15.9%). [5] In the present study, we focused on HCC patients with pelvic bone metastases because only a few case studies have evaluated radiotherapy for pelvic metastases, [12] and because there exists no consensus about target volume and radiation dose for HCC pelvic bone metastases. When the pelvic bone is categorized into the three parts, the sacroiliac joint lumbar spine (5.9%) is the most frequent site of pelvic bone metastasis, followed by the ilium (4.0%) and the sacrum (3.5%). [5]

### Clinical results from EBRT dose-response relation and toxicity

He *et al.* reported that partial pain relief was achieved in 70.2% of patients (144 of 205 patients), complete pain relief was achieved in 29.3% (60 patients), and overall pain improved in as much as 99.5% of HCC patients.[5] There was no dose-response relationship for palliation of bone metastasis, but the retreatment rate was higher in patients with expansile soft tissue. In this study, 40% of HCC bone metastases showed hypervascular soft-tissue mass.[5] Concerning pelvic bone marrow, several studies have evaluated the dose-volume effects of pelvic bone marrow radiotherapy in rectal and cervical cancer patients. Mell *et al.* contoured pelvic bone marrow (BM) and evaluated dosimetric parameters associated with acute hematologic toxicity in cervical cancer patients undergoing concurrent chemotherapy and intensity-modulated pelvic radiotherapy.[13] Low pelvic bone marrow dose was associated with increased Grade II or worse leukopenia and neutropenia (*p*=0.006 and *p*=0.037, respectively). Mell *et al.* suggested that even low dose irradiation to the pelvic bone frequently induces hematologic toxicity and that the volume of irradiated pelvic bone marrow is important. Lee *et al.* also described the volume of red bone marrow as an independent risk factor associated with hematologic toxicity (*p*=0.014).[14] The present study evaluates pelvic bone radiotherapy for pelvic bone metastasis. Determining the target volume is very important in these patients because pelvic bone marrow volume is critical for hematologic toxicity.[14] P53 has been suggested to play a key role in determining how a cell responds to RT. Gomes *et al.* reported that irradiation induced a decrease in cell survival when P53 was overexpressed *in vitro*.[15] We were unable to analyze P53 expression owing to the retrospective nature of this study.

Grade IV neutropenia and thrombocytopenia (n = 4 and n = 3, respectively) were more frequently observed with compartmental target volume treatment than with marginal target volume. Therefore, we suggest that treatment volume should be decided according to bone marrow volume and other bone involvement. One case of isolated metastatic HCC arising from the pelvic bone has been described: a 73-year-old man presented with a large mass on the pelvic bone (13 × 10 cm). He was treated with radiotherapy and transarterial chemoembolization to the pelvic bone followed by chemotherapy, resulting in near complete tumor regression.[12]

### Combination of sorafenib and radiation therapy

There is little information about the combined radiotherapy and sorafenib for bone metastases in patients with hepatocellular carcinoma. In our previous report, the feasibility of sorafenib combined with radiation therapy in 18 patients with hepatocellular carcinoma was evaluated.[16] The in-field response rate was 100% in the primary group and 60% in the measurable metastasis group. Toxicities of grade 3-4 related combined treatment were duodenal bleeding in 1 (6%) patient and elevation of aspartate transaminase in 1 (6%) patient. In recent phase II study, the tolerance and toxicities of conventionally fractionated radiation therapy (2-2.5 Gy per fraction; dose range 40-60 Gy) with sorafenib were evaluated.[17] Hepatic toxicities were major determinant of the combined treatment in this study as follows, six patients (15%) developed treatment-related hepatic toxicity grade ≥ 3, and 3 of them were fatal. Brade et al. reported the phase I study of the combination of sorafenib with stereotactic body radiation therapy for hepatocellular carcinoma.[18] In this study, clinical dose-limiting toxicities (grade 3 large bowel bleeding and grade 4 bowel obstruction) were noted dependent on the irradiated volume and dose of sorafenib. Therefore, the combination should be used with caution and recommended with a clinical trial.

### Clinical target volume of pelvic bone metastasis from HCC

Metastatic lesions are typically osteolytic, although sclerotic lesions can be encountered from primary tumors such as prostate or breast carcinoma.[19] He *et al.* reported that purely osteolytic lesions were present in 2.4% of 205 HCC patients with bone metastasis.[5] Most lesions (97.6%) had a combination of both osteolytic and osteoblastic components. For pelvic bone HCC metastases in the present study, only 7.1% were pure osteolytic lesions, and 28.1% had soft tissue extension. Although the number of pure osteolytic lesions was small (n = 10), local control and pain control were quite poor (29% and 14%, respectively) when such lesions were treated with marginal target volume. Therefore, we propose that compartmental treatment should be considered in these patients.

For target volume delineation, some guidelines and clinical recommendations exist for spine metastases in metastatic breast cancer patients. For example, CTV should include the complete vertebral body in cases of vertebral involvement.[20] Although the pelvis is a typical metastatic site, little consensus exists regarding target volume delineation for radiotherapy of pelvic bone metastases. Because the pelvis has a curved shape and encompasses bowel loops and the bladder, contouring the target volume and protecting the organs at risk is challenging. In the present study, target volume encompassing the gross tumor plus a wide margin encompassing pelvic bone marrow showed significantly improved local control as compared to clinical target volume plus the margin of the involved metastatic bone tumor (*p*=0.001), without significantly increasing toxicity.

Recent technological advances, such as intensity modulated radiotherapy and stereotactic body radiotherapy, have enabled more successful radiation treatment by delivering a substantial dose of radiation to the tumor and sparing healthy normal tissues.[21] Although many studies have evaluated clinical features and radiotherapeutic strategies in patients with spinal bone metastasis, few studies have been performed for HCC patients with pelvic bone metastasis. Although the present study is a retrospective analysis, the results of this study provide practical guidelines for radiation treatment for HCC patients with pelvic bone metastasis. Future clinical trials will focus on comparing treatment results using different target volumes, or different treatment strategies.

In conclusion, radiotherapy for pelvic bone metastases provides considerable pain palliation and local control for HCC patients. Treatment of the entirety of the involved bone was associated with improved local control rates and local failure-free survival. High-dose radiation was associated with an improved treatment response.

## PATIENTS AND METHODS

### Patient selection

The medical records of HCC patients who underwent RT for bone metastasis between 2005 and 2011 were retrospectively reviewed. Eligibility criteria for this study were: (1) age ≥ 18 years; (2) an initial diagnosis of primary HCC; (3) a diagnosis of bone metastasis; (4) the presence of pelvic bone metastasis, which includes the sacrum, iliac bone, pubic bone, ischium, and acetabulum; and (5) radiation dose > 20 Gy in a EQD2 assuming an α/β ratio of 10 Gy. A diagnosis of bone metastasis was based on the presence of symptoms and radiologic imaging studies. Radiologic imaging studies included computed tomography (CT), magnetic resonance imaging (MRI), and whole body bone scan (WBBS). CT or MRI was considered confirmatory to determine the presence of soft-tissue extension with bone destruction and the extent of osteolytic or osteoblastic metastasis. Histologic confirmation of bone metastasis was not mandatory in this study. We analyzed 146 cases from 89 patients with pelvic bone metastasis from HCC treated with RT.

Metastasis characteristics were categorized as either pure osteolytic or mixed (osteolytic and osteoblastic). Pure osteolytic metastasis was defined as the presence of bone destruction with no new bone formation, as detected by CT or MRI, as well as no increased hot uptake during the WBBS. Mixed metastasis was defined as new bone formation in the involved bone that was visible by CT or MRI as well as increased hot uptake during the WBBS.

### Treatment

**Figure 3:**
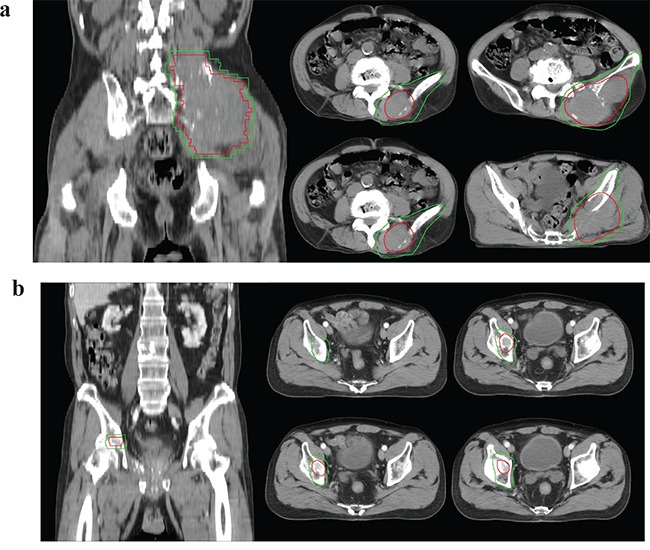
Compartmental target volume and marginal target volume **a.** Compartmental target volume for iliac bone metastasis. A 72-year-old man had a left iliac bone metastasis with soft tissue extension. He was treated with compartmental target volume and a dose of 40 Gy in 16 fractions. **b.** Marginal target volume for acetabulum bone metastasis. A 68-year-old man had a right acetabulum metastasis. He received 45 Gy in 15 fractions with marginal target volume. Gross tumor volume (red line), Clinical target volume (green line).

Planning CT was performed on patients in a supine position. We defined gross tumor volume (GTV) as the volume of the metastatic tumor detected on CT or MRI. We defined CTV as follows: compartment target volume, which encompassed the gross tumor plus a wide margin considering pelvic compartments; or marginal target volume, which encompassed the gross tumor plus adequate margin. Detailed explanations of compartment target volumes are as follows: (1) when a metastatic lesion was present in the sacrum, compartment target volume encompassed the whole sacrum; (2) when a metastatic lesion was present in the iliac bone, compartment target volume encompassed the entire iliac bone including the iliac wing; (3) when a metastatic lesion was present in the pubic bone, ischium or acetabulum, compartment target volume encompassed the entirety of the pubic bone, ischium, and acetabulum. GTV, compartment target volume, and marginal target volume are shown in Figure 3 and Supplementary Figure 1. Figure 3a shows a patient treated with compartmental target volume. A 72-year-old man had metastasis to the left iliac bone with soft tissue extension. He was treated with three-dimensional conformal radiotherapy (3D-CRT) at a dose of 40 Gy in 16 fractions. Pain was decreased according to the visual analogue scale (VAS) from 6 to 2, and the metastatic lesion was controlled until his 10-month follow-up examination. Figure 3b show a patient treated with marginal target volume. A 68-year-old man had a right acetabulum metastasis. He received 45 Gy in 15 fractions using 3D-CRT. His pain was decreased from VAS 8 to 3, but a right pubic bone metastasis at the margin of the initial RT field was discovered at his 18-month follow-up examination. After local failure, he received re-irradiation with compartmental target volume encompassing entire lower pelvic bone. Supplementary Figure 1 shows GTV, compartmental CTV, and marginal CTV. Supplementary Figures 1a, b, and c show target volumes for sacrum, iliac, and lower pelvic bone, respectively. We selected a patient without bone metastasis and delineated target volumes to show the difference between different CTVs. Seventy-eight (53%) of 146 cases were treated with compartment target volume, and the remaining 68 (47%) cases were treated with marginal target volume. CTV was modified to reduce the dose to organs at risk (OARs), such as the small bowel, bladder, rectum, and anus. For planning target volume (PTV), a 0.7 cm margin was applied to CTV considering patient movement and daily setup error.

Sixteen patients (11%) were treated with two-dimensional RT, 119 (82%) with 3D-CRT using 6 MV photons generated from a linear accelerator, and 11 (7%) with intensity-modulated radiotherapy (IMRT) using tomotherapy. The median RT dose was 35 Gy (range, 17.5-60 Gy), and the median fractional dose was 3 Gy (range, 2.5-6 Gy). The most commonly used RT schedule was 30 Gy in 10 fractions.

### Evaluation

The Child-Pugh classification is calculated from 5 subscores: 3 objective clinical laboratory values (total bilirubin, serum albumin, and international normalized ratio) and 2 subjective variables (the severity of ascites and hepatic encephalopathy). Each variable is scored 1-3, with 3 indicating the most severe derangement. A total score of 5-6 points is classified as A, 7-9 points is classified as B, and 10-15 points is classified as C.

The pain response was assessed as a subjective pain response using the VAS. VAS is used to describe the intensity of pain or how much pain the patient reports feeling., The patient is asked to identify how much pain he or she is feeling on a numeric rating scale from 0 (no pain) to 10 (the worst pain that he or she can imagine). Patients were asked to report the level of pain before, immediately after, and 1 month after treatment. A positive response was defined as a ≥ 50% reduction in the VAS numerical pain rating. Responses were evaluated for a total of 1 month, beginning with the first day of treatment.

Treatment-related toxicities were assessed at every follow-up examination. Toxicity was monitored according to the Common Terminology Criteria for Adverse Events v3.0 (CTCAE v3.0), through a physical examination and laboratory testing for levels of hemoglobin, white blood cells, and platelets, both during RT treatment and after completion of the RT schedule.

### Statistical analysis

The primary endpoint of this study was LCR. Local failure was defined as recurrence of the tumor within the RT filed of a treated bone at any point after RT. Overall survival (OS) was calculated from the date of RT inception to the date of death or the last follow-up. The PFI was calculated from the date of RT inception to the date of pain recurrence or the last follow-up. OS, LFFS, and PFI were calculated via the Kaplan–Meier method using a log-rank test to estimate statistically significant differences. Factors affecting OS, LFFS, and PFI were identified using a Pearson's *χ^2^* test or a Fisher's exact test. Continuous variables were analyzed using a Mann-Whitney *U* test and a Student's *t*-test. We considered *p* values <0.05 significant. For all analyses, SPSS version 20.0.0 (IBM Corporation; Armonk, NY, USA) was used.

## SUPPLEMENTARY MATERIALS FIGURES AND TABLES


