# Supplementary material for: High dose and compartmental target volume may improve patient outcome after radiotherapy for pelvic bone metastases from hepatocellular carcinoma
Source: Oncotarget. 2016 Jun 1;7(33):53921–9. doi: 10.18632/oncotarget.9767 (PMC5288232; doi:10.18632/oncotarget.9767)
Supplement: Supplementary file 1 [file oncotarget-07-53921-s001.pdf]

# High dose and compartmental target volume may improve patient outcome after radiotherapy for pelvic bone metastases from hepatocellular carcinoma

## SUPPLEMENTARY FIGURE AND TABLES

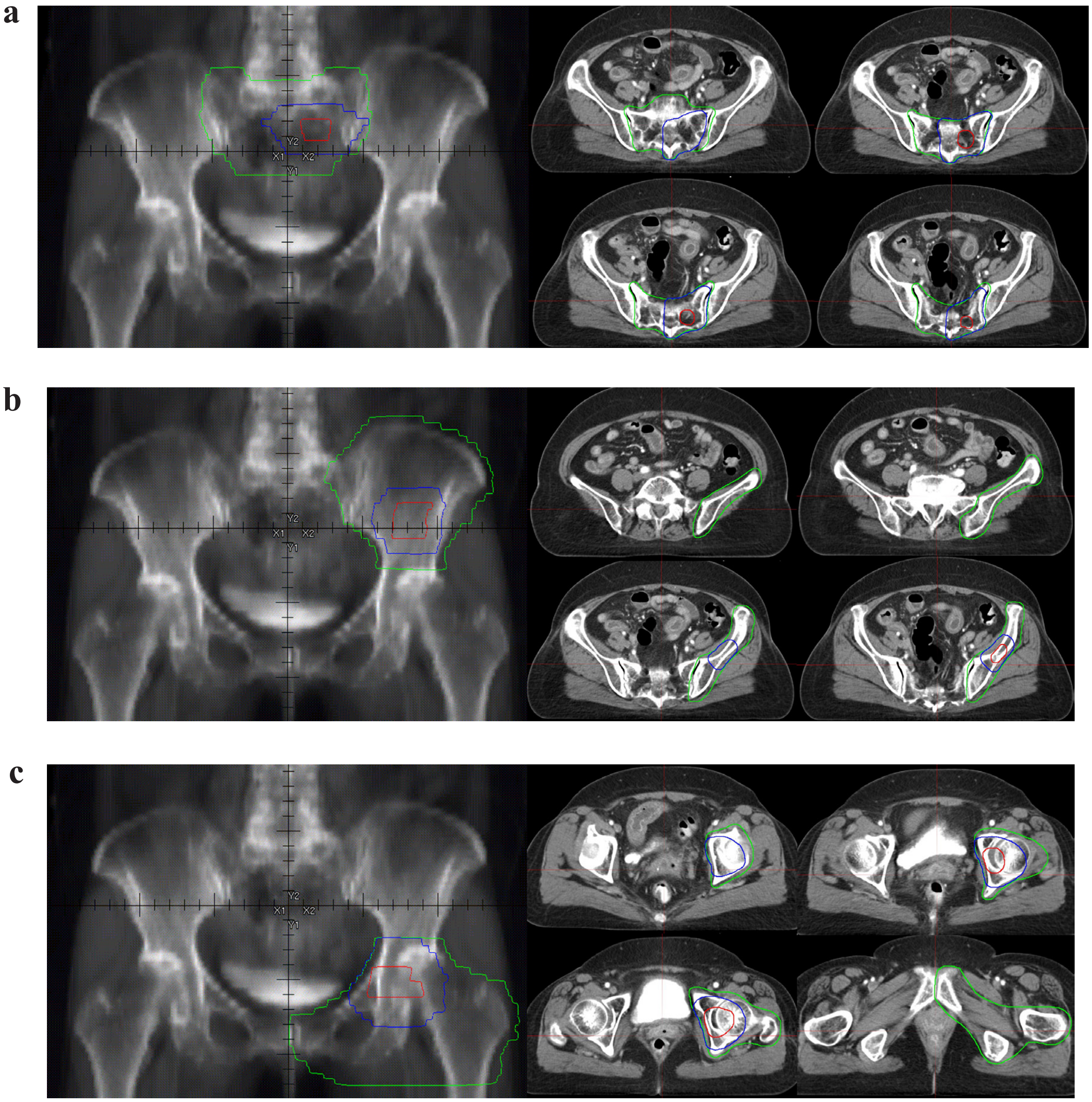

**Supplementary Figure S1: Compartmental target volume and marginal target volume.** Sacrum **a.** ilium **b.** and lower pelvic bone **c.** Gross tumor volume (red line), compartment target volume (green line), and marginal target volume (blue line).

**Supplementary Table S1: Patient characteristics**

| Variable | Group | n | % |
|---|---|---|---|
| Age (years) | Median | 56 | |
| | Range | 36-79 | |
| Sex | Male | 70 | 79% |
| | Female | 19 | 21% |
| Virus | HBV | 77 | 86% |
| | HCV | 7 | 8% |
| | NBNC | 5 | 6% |
| Child-Pugh classification | A | 71 | 80% |
| | B | 11 | 12% |
| | C | 7 | 8% |
| Primary tumor control | Controlled | 65 | 72% |
| | Uncontrolled | 24 | 28% |

Abbreviations: HBV, Hepatitis B Virus; HCV, Hepatitis C Virus; NBNC, Non-B Non-C.

**Supplementary Table S2: Pain control rate according to target volume**

| Variable | Group | Pain control rate (%) | | *p* value |
|---|---|---|---|---|
| | | Compartmental | Marginal | |
| Pelvic subsite | Sacrum (44) | 59% | 80% | 0.283 |
| | Ilium (43) | 82% | 64% | 0.171 |
| | Acetabulum (26) | 86% | 67% | 0.198 |
| | Pubic bone (17) | 87% | 57% | 0.16 |
| | Ischium (16) | 78% | 25% | 0.077 |
| Site categorization | Upper pelvic bone (87) | 68% | 68% | 0.954 |
| | Lower pelvic bone (43) | 79% | 57% | 0.077 |
| Primary control | Controlled (36) | 57% | 62% | 0.769 |
| | Uncontrolled (110) | 77% | 65% | 0.138 |
| No. of pelvic bone metastasis | Single (59) | 72% | 70% | 0.826 |
| | Multiple (87) | 71% | 61% | 0.285 |
| Other bone involvement | Present (110) | 70% | 64% | 0.507 |
| | Absent (36) | 79% | 65% | 0.463 |
| Soft tissue extension | Present (41) | 84% | 63% | 0.15 |
| | Absent (105) | 67% | 64% | 0.812 |
| Metastasis characteristic | Mixed (136) | 73% | 70% | 0.722 |
| | Pure osteolytic (10) | 33% | 14% | 1 |
